# Improving accessibility of EPR-insensitive tumor phenotypes using EPR-adaptive strategies: Designing a new perspective in nanomedicine delivery

**DOI:** 10.7150/thno.37204

**Published:** 2019-10-17

**Authors:** Alexander Dhaliwal, Gang Zheng

**Affiliations:** 1 Department of Medical Biophysics, University of Toronto, Toronto, ON Canada; 2 MD/PhD Program, Faculty of Medicine, University of Toronto, Toronto, ON Canada; 3 Princess Margaret Cancer Centre, University Health Network, Toronto, Canada; 4 Institute of Biomaterials and Biomedical Engineering, University of Toronto, Toronto, ON Canada

**Keywords:** EPR-insensitive tumor phenotypes, EPR-adaptive strategies, nanomedicine

## Abstract

The enhanced permeability and retention (EPR) effect has underlain the predominant nanomedicine design philosophy for the past three decades. However, growing evidence suggests that it is over-represented in preclinical models, and agents designed solely using its principle of passive accumulation can only be applied to a narrow subset of clinical tumors. For this reason, strategies that can improve upon the EPR effect to facilitate nanomedicine delivery to otherwise non-responsive tumors are required for broad clinical translation. EPR-adaptive nanomedicine delivery comprises a class of chemical and physical techniques that modify tumor accessibility in an effort to increase agent delivery and therapeutic effect. In the present review, we overview the primary benefits and limitations of radiation, ultrasound, hyperthermia, and photodynamic therapy as physical strategies for EPR-adaptive delivery to EPR-insensitive tumor phenotypes, and we reflect upon changes in the preclinical research pathway that should be implemented in order to optimally validate and develop these delivery strategies.

## 1. Introduction

One of nanomedicine's primary contributions to cancer research is its illustration of the power of intentional agent design as a tool for enhancing drug delivery. This philosophy has spurred decades of research into nanoparticle formulations and functionalization of existing drugs, pushing forward the concept of personalizing medical treatment by tailoring drug design for specific applications. The coming years hold the potential to revolutionize treatment options for a variety of ailments, chief among them being solid tumor cancers.

In the wake of the massive intellectual and financial investment in nanomedicine design, scrutiny of the founding principles and assumptions upon which the discipline has been built is warranted. Few would argue that that the most prevalent nanomedicine designs principle in cancer treatment is the idea of the Enhanced Permeability and Retention (EPR) effect. First introduced by Matsumura and Maeda in the 1980's 1, the EPR effect describes the phenomenon of high molecular weight drug and nanomedicine accumulation preferentially inside solid tumor models versus healthy tissue counterparts. This was primarily attributed to two tumor characteristics: a) a leaky tumor vasculature as a result of the accelerated angiogenesis that is a hallmark of cancer and b) impaired lymphatic drainage, another result of the disorganized growth of tumors. These original studies showed dramatic uptake in tumor tissue, displaying elevated drug concentrations that increased up to 72 hours after injection with minimal accumulation in healthy tissue. The theory did face some criticism, most prominently via work by Jain *et al.* in their discovery that elevated interstitial fluid pressure (IFP) and heterogeneous blood supply limited macromolecular delivery to tumors 2,3. Nevertheless, its basis and implications remain largely unchanged more than three decades later.

The observation of the EPR effect provided a clear design philosophy for cancer therapy development - increase drug concentration at the site of interest relative to healthy tissue as a means of alleviating treatment side effects. This was explored mainly through optimizing formulations for increased blood circulation time, such as encapsulation of agents within liposomes and surface chemistry modification using PEG chains. These changes led to longer exposure of the tumor site to circulating nanoparticles, increasing relative accumulation. Time-dependent optimization was coupled with careful size selection of agents, which were chosen to act optimally for slow renal excretion, low off-target liver uptake, and maximal tumor uptake. Many of the first and arguably most successful clinically approved nanoformulations, including Doxil® and Abraxane®, capitalize on these principles. While these drugs provide clinical benefits by means of reduced toxicity, the high level of specificity and sensitivity seen by Maeda and Matsumura's early work has not be recapitulated to the same degree in a clinical setting 4,5,6.

This unrealized potential has led to a questioning of the ubiquity of the EPR effect, including critiques by its founders 7,8. This was partially driven by work that showed high heterogeneity both within and between spontaneous tumor types. In a clinical study, Harrington *et al.* showed accumulation of radiolabelled PEGylated liposomes ranging from as low as 2.7% ID/g in ductal breast cancer to as high as 53.0% ID/g in head and neck cancers, with other spontaneous lung, brain, and cervical cancers spanning this range 9. More recently, Hansen *et al.* showed increased accumulation in carcinomas relative to sarcomas in spontaneous canine solid tumors 10. These examples demonstrate that the EPR effect cannot be considered a universal feature of all solid tumors.

To overcome these limitations in achieving consistent delivery to a varied clinical target, many researchers are exploring a more inclusive nanomedicine design philosophy - delivery strategies that do not rely on the fixed, passive accumulation capacity inherent to a given tumor. Such strategies complement the EPR effect by modifying tumor accessibility and susceptibility in order to increase nanomedicine delivery across many solid tumor phenotypes, thereby maximizing their clinical applicability. This review hopes to provide a timely overview of the current strategies that fall under this classification of EPR-adaptive delivery, and it seeks to critically compare the advantages and challenges inherent to the associated design processes. This includes a review of the translational potential of these design strategies - both in a clinical and interdisciplinary sense - as well as an identification of the necessary pre-clinical tools required to effectively evaluate EPR-adaptive delivery strategies.

## 2. Design and Delivery Philosophies

### 2.1 EPR-based Delivery

The passive nature of the EPR effect means that it has the capacity to affect the biodistribution of all nanosized agents for cancer targets. In this manner, it is true that all nanomedicine delivery strategies benefit in some form from the EPR effect, albeit to varying degrees across different tumor types. EPR-based delivery can be conservatively thought of as a delivery strategy that does not attempt to modify inherent tumor accessibility in an effort to increase agent accumulation and improve therapeutic potential.

This design philosophy places focus upon modification to nanomedicine formulation to improve passive accumulation of the agent, primarily by increasing circulation time and optimizing nano-bio interactions, for example through size selection. Beyond static designs, formulations can also be chemically functionalized to actively target biochemical signatures of a tumor 11 or exploit endogenous stimuli to improve uptake and activity *in situ* 12. While these areas have seen significant progress, they suffer from the same limitation on generalizability. Because their efficacy is intrinsically tied to the characteristics of tumors and passive accumulation, these nanomedicine designs will lead to variable uptake and efficacy when moved to the clinic.

What has been identified as one of the primary detractors of the clinical success of the traditional EPR-based delivery strategy is the over-representation of the EPR effect in preclinical models of cancer, most notably subcutaneous xenografts. Heneweer *et al.* illustrated that three different prostate cancer cells lines from vastly different tumor phenotypes all displayed similar longitudinal accumulation of radiolabeled albumin when xenografted into mice, all reaching 5% ID/g 13. This makes it harder to accurately extrapolate about how an improvement in a preclinical model will translate to clinical tumors. Thus, for traditional EPR-based strategies to persist, they must be selectively applied to specific tumor phenotypes.

**Figure 1:**
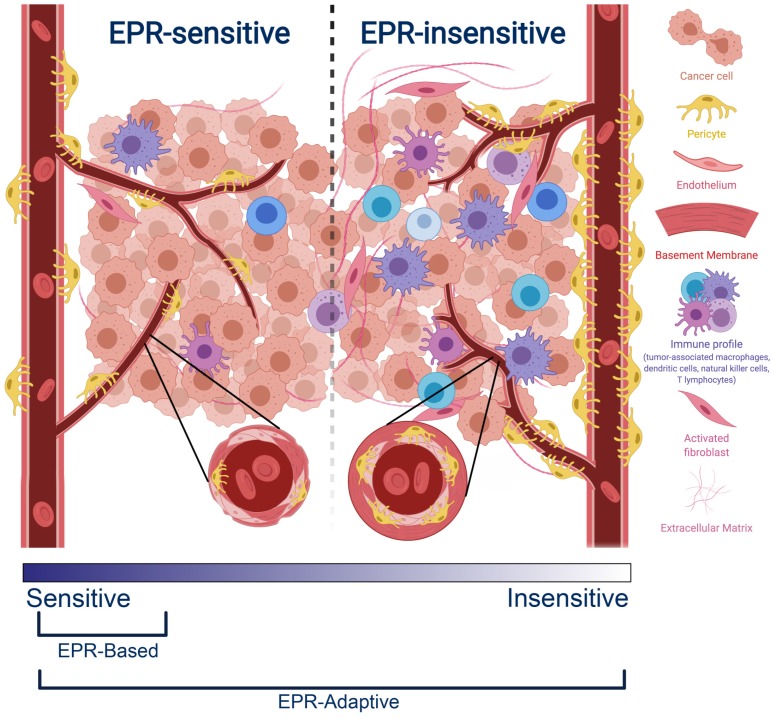
Comparison between an EPR-sensitive tumor phenotype (typical of preclinical cancer models) and an EPR-insensitive tumor phenotype (typical of clinical human tumors). EPR-sensitive tumors are characterized by a hyperpermeable vasculature with large endothelial fenestrations, uniformly low pericyte coverage, a relatively sparse extracellular matrix, and a small immune profile. In contrast, the EPR-insensitive phenotype has a more well-developed and branched vasculature, smaller endothelial fenestrations, heterogeneously high or low pericyte coverage, a relatively dense extracellular matrix, and a more developed immune profile. These characteristics exist on a spectrum - EPR-based delivery strategies operate best on a subset of EPR-sensitive tumors, whereas EPR-adaptive delivery strategies are designed to function across this spectrum.

It is clear that there exists a phenotypic spectrum for tumors with regards to how treatable they are under an EPR-based delivery strategy, with preclinical models exhibiting more sensitivity and clinical models showing more insensitivity (Figure 1). The specific characteristics that confer EPR-sensitivity to a tumor are hypothesized to include a hyperpermeable and fenestrated vasculature, low pericyte coverage, a sparse ECM, and a muted immune profile, the evidence for which is described later in this review. Attempts to improve the efficacy of existing nanomedicines may be aided through targeted selection of patients with tumor phenotypes most sensitive to traditional EPR-based therapy. Some preclinical measures of vascular function have been shown to be correlated with EPR-sensitivity 14,15,16, but clinical studies are still early in their interpretation of these relationships 17,18. Thus, while these efforts may eventually help to determine the target populations most receptive to EPR-based nanotherapy, they do not provide a clear path towards the treatment of EPR-insensitive individuals.

### 2.2 EPR-adaptive Delivery

While the traditional EPR-based design philosophy has led to the clinical success of some formulations, it reneges on the promise for nanomedicine to be Ehrlich's “magic bullet”, capable of selectively targeting tumors across a wide range of cancer phenotypes. Alternative drug delivery strategies that forego the requirement of an EPR-sensitive tumor phenotype do exist, and they have been receiving increased attention in recent years as disenchantment with the EPR effect grows. This class of techniques, herein referred to as **EPR-adaptive delivery**, is characterized by its ability to modify tumor accessibility in an effort to increase agent delivery and therapeutic effect. While the EPR effect itself may still be partially responsible for the overall delivery efficacy of certain agents developed under this principle, these EPR-adaptive strategies are generalizable to a broader range of clinical targets, including those historically inaccessible to traditional EPR-based strategies. In alleviating the dependence of these strategies on the EPR effect as a primary determinant of delivery success, these approaches overcome the pitfalls commonly attributed to conventional nanomedicine delivery.

EPR-adaptive delivery encompasses both chemical and physical techniques used to improve nanomedicine biodistribution. Chemical mediators seek to improve tumor access through direct modification of the tumor vessels, stroma, and cancer cells. Vascular agents, such as bradykinin, prostaglandins, and nitrous oxide, induce an inflammation-like state to restore tumor blood flow and improve tumor influx, thus boosting permeability to circulating drugs 11,19,20,21,22,23,24,25,26. Beneficial vascular changes also include vascular normalization, a technique whereby tortuous, insufficient tumor vasculature is functionally restored using mild anti-angiogenic therapy, which has been shown to improve nanomedicine delivery and improve therapeutic outcomes 27,28. Treatment with enzymes that digest the physical structure of the ECM, such as collagenase 29 and hyaluronidase 30,31,32,33, lower interstitial fluid pressure in tumors, while direct induction of apoptosis in cancer and stromal cells can reduce pressure on microvasculature to improve depth of penetration of agents into tumors 34,35. Hormonal effectors, such as the angiotensin II receptor antagonist losartan, can capitalize on these principles as well - in this case, its action decompresses tumor vessels, increases tumor perfusion, and suppresses collagen synthesis, leading to enhanced drug accumulation 36,37. This has shown clinical viability as a combination neoadjuvant therapy with FOLFIRINOX (fluorouracil, leucovorin, oxaliplatin, and irontecan) in a Phase II clinical trial of 49 patients with locally advanced pancreatic cancer, displaying improved margin-negative resection rates and prolonged survival 38. These chemical strategies provide the attractive ability to improve the therapeutic effects of existing nanomedicines in a manner that is cohesive with the current clinical workflow of combination therapy. While these fall under the classification of EPR-adaptive delivery, they have been extensively reviewed elsewhere 7,20,39,40,41,42,43, and thus will not be the focus of this review.

Physical techniques represent the other arm of the EPR-adaptive delivery strategy. These involve the use of external stimuli to modify a delivery site either prior to or concurrent with nanomedicine delivery. This includes radiation, ultrasound, hyperthermia, and photodynamic therapy, and these strategies will be the focus of discussion in this review. Each of these capitalize on unique physiological responses to improve agent uptake and augment retention in a manner that is applicable to a majority of solid tumor phenotypes (Table 1).

#### 2.2.1 Radiation

Given the common usage of radiotherapy with adjunctive chemotherapy in cancer treatment, it is only natural that one of the first physical modalities used to enhance nanomedicine delivery was radiation. Traditional radiotherapy delivers ionizing radiation to the tumor site under image guidance, leading to damage at tumor areas and minimal dosing of neighbouring healthy tissue. This has a variety of effects on cancer, endothelial, and stromal cells, including increased hypoxia, decreased interstitial fluid pressure, and lowered solid tumor pressure 44, all of which can impact the delivery of circulating nanomedicines.

Li *et al.* conducted some of the original studies in this area combining nanoformulations of chemotherapy and radiation pre-treatment. They showed that ionizing radiation delivered 24 hours before injection of PG-TXL had a 4.4-fold enhancement of tumor growth delay compared to PG-TXL treatment alone in ovarian OCa-1 intramuscular tumors, extending survival time by 46 days 45. de Lange Davies* et al.* conducted comparable studies using liposomal doxorubicin as a model agent and radiation delivered to osteosarcoma tumors 24 hours post-treatment. They showed a 2-4-fold improvement in nanoparticle uptake in both xenograft and orthotopic tumors when either single or fractionated-dose ionizing radiation was administered 46. Moderate efficacy in patients has been shown through multiple phase I and II clinical trials using various liposomal chemotherapy formulations that, alongside radiation treatment, increased agent accumulation, reduced tumor burden, and prolonged survival in patients with lung cancer 47,48,49,50, head and neck cancer 47,48,51, breast cancer 52, and sarcomas 53, all while showing improved tolerance compared to radiochemotherapy 54,55.

The ability of radiotherapy to decrease intratumoral pressure is an asset to its ability to enhance nanomedicine delivery. Reductions in pressure are due to a combination of factors, including vascular regression, death of tumor cells which compress microvasculature, and decreased resistance to blood flow 56,57. Collectively, these allow for more convective movement towards the tumor core, which is believed to be a relevant mechanism for nanomedicine transport 2,3. This was another finding highlighted in de Lange Davies *et al.*'s seminal paper, as they showed that liposomal doxorubicin penetrated more deeply in both xenograft and orthotopic tumors when combined with radiation therapy. Tumor histology revealed that, while accumulation in untreated tumors was largely perivascular, irradiated tumors showcased a more uniform intratumoral drug distribution 46. Attempts to image IFP following radiation treatment have shown similar findings, showing that both increased perfusion and decreased IFP are spatially correlated with areas in which liposomes preferentially accumulate in tumors 58. This illustrates that radiation not only increases bulk tumor uptake, it also improves the spatial distribution of the administered agents, increasing the likelihood that they reach their intended cellular targets.

However, the efficacy of radiotherapy to improve drug delivery has strong temporal and spatial dependence. The remodelling that occurs in tumors in response to fractionated radiation treatment can lead to upregulation of the ECM, and new vessel growth may exacerbate tumor hypoxia and lead to insufficient perfusion for delivery 44,59. Thus, most studies exploring radiochemotherapy with nanomedicines highlight the importance of optimizing radiation dose and fractionation schedule in order to maximize delivery during highly perfusive, susceptible tumor states. Overall, this makes radiation an appealing option for conjunctive therapy and delivery enhancement; however, its success in improving nanomedicine tumor accumulation is predicated on balancing its beneficial capacity to alleviate intratumoral pressure and its detrimental effect on perfusion reduction.

#### 2.2.2 Ultrasound-assisted delivery

**Figure 2:**
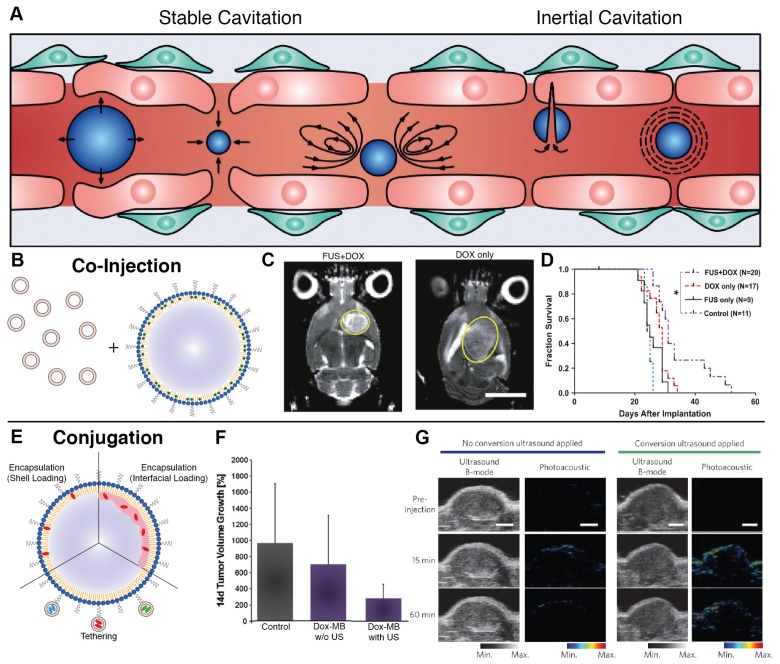
A. Cavitation-based mechanisms by which ultrasound can enhance nanomedicine delivery, including transient disruption through stable cavitation and destructive opening through inertial cavitation (adapted from 157). B. Co-injection involves simultaneous administration of microbubbles and nanoparticles with ultrasound priming to improve delivery. C-D. Application of microbubble-enhanced focused ultrasound and pre-administered liposomal doxorubicin on rat brains bearing 9L glioma xenografts results in tumor size reduction and prolonged survival compared to doxorubicin only controls (adapted from 75). E. Conjugation encompasses both nanoparticle tethering and agent encapsulation to produce a highly co-localized platform for delivery improvement. F. The combination of doxorubicin-loaded microbubbles and ultrasound achieve enhanced therapeutic reduction in DSL6A pancreatic xenograft size compared to drug-loaded bubbles alone (adapted from 90). G. *In situ* microbubble-to-nanoparticle conversion of porphyrin-lipid microbubbles upon ultrasound irradiation leads to enhanced nanoparticle delivery as measured by photoacoustic imaging (adapted from 156).

Ultrasound is an incredibly versatile clinical modality, seeing universal applicability as a diagnostic imaging technique due to its high tissue penetration depth (~10 cm at 1 MHz), non-invasiveness, and ease of use. To improve contrast of vessels during imaging, gas-filled agents called microbubbles have long been used, as they produce non-linear oscillations called cavitations that dramatically increase contrast between vascularized structures. Beyond imaging, these oscillations can be exploited in two primary ways - at low intensities, microbubbles undergo stable cavitation, leading to transient disruption of endothelium and an increase in vascular permeability. At high intensities, microbubbles undergo inertial cavitation, creating shockwaves that can perforate cell walls and lead to elevations in temperature and reactive oxygen species. While the latter can be exploited for therapeutic delivery with high-intensity focused ultrasound, the former is ideal for invoking biomechanical effects that transiently disrupt vascular endothelium to increase local drug extravasation (Figure 2A) 60,61.

The efficacy of pre-treatment with ultrasound and microbubbles in improving nanoparticle delivery is observed across both *in vitro* cell lines and *in vivo* animal models, suggesting that the mechanism involves both paracellular (i.e., disruption of tight junctions) and transcellular (i.e., transcytosis) transport 62,63,64. Both mechanisms benefit from co-localization of the disruptive process and the nanomedicine to be delivered. Through tuning of the acoustic pressures generated by ultrasound, vascular permeability can be enhanced in a variety of tissues, including a variety of tumor types 65,66,67,68 and traditionally impermeable sites such as the brain 69,70,71,72,73. Hynynen *et al.* pioneered this application for notoriously inaccessible brain tumors. Their application of MRI-guided focused ultrasound improved liposomal doxorubicin accumulation in healthy brains in a manner linearly dependent on microbubble dosage, showing a 3.5-fold increase in delivery with no surrounding tissue damage 74. When moved to a rat 9L gliosarcoma model, treatment enabled a 24% increase in median survival time compared to non-treated rats (Figure 2C-D) 75. An ongoing clinical pilot seeks to better understand whether or not such improvements will be conserved in clinical models of glioblastoma 76.

The most common delivery strategies employed with ultrasound are co-injection and conjugation. Co-injection involves injection of free nanoparticles either preceding, following, or concurrent with ultrasound treatment, the latter of which would involve mixing of the individual agents immediately prior to injection (Figure 2B) 77. This allows for users to leverage clinically-available microbubbles, permits simple clinical dose customization, and presents a clear pathway towards clinical translation, especially if utilizing agents which have already received clinical approval for their individual use. Co-injection has shown considerable success in pre-clinical xenograft models across a variety of tumor phenotypes 78. Theek *et al.* showcased that two EPR-insensitive phenotypes (highly cellular A431 epidermal xenografts and highly stromal BxPC-3 pancreatic carcinoma xenografts) showed increased accumulation of liposomes after ultrasound irradiation compared to untreated controls 66. The impact of size upon uptake was explored by Lin *et al.,* where uptake of lipid-coated CdSE quantum dots with sizes ranging from 30 nm to 180 nm in CT-26 colorectal adenocarcinoma xenografts was measured after pre-treatment with ultrasound and Sonovue® microbubbles. This pre-treatment strategy showed that the greatest increases in extravasation were achieved with the smaller, 30 nm nanoparticles 79. This promise has translated well to clinical trials: Dimcevski *et al.* have shown that low-intensity ultrasound enabled with Sonovue® microbubbles and pre-administered gemcitabine nearly doubled median survival of patients with inoperable pancreatic ductal adenocarcinoma compared to gemcitabine alone 60.

In contrast, conjugation loads the nanoparticle directly onto the microbubble for maximum co-localization. It encompasses both encapsulation of nanoparticles within the microbubble and chemical tethering of agents to the microbubble shell (Figure 2E) 80. While encapsulation is largely limited to hydrophobic loads that can sequester within the microbubble shell or sit at the shell-air interface 81,82, tethering allows for attachment of almost any agent using reliable linking strategies such as biotin-streptavidin binding 83,84,85,86 or click chemistry 87,88 and versatile carriers such as liposomes 85,89. Tinkov *et al.* were one of the first groups to fabricate microbubbles with liposomal doxorubicin and showed a 12-fold increase in drug accumulation in DSL6A murine pancreatic xenografts compared to non-irradiated tumors in a bilateral tumor model (Figure 2F) 90. Similarly, Yan *et al.* showed a 4.31-fold increase in liposomal paclitaxel uptake in a bilateral 4T1 breast cancer model 86. Beyond drug delivery, numerous nanoparticle contrast agents for MRI 91,92,93,94, PET 95, and fluorescence 96,97,98 imaging have also been conjugated to microbubbles to develop multimodal agents, showing the capacity for functionalization as a means to elevate the utility of clinical ultrasound.

In comparison to co-injection, conjugation does not require *ex vivo* mixing before administration, allows for ratio-metric loading of nanoparticles to microbubbles to optimize co-delivery, and may hold greater potential for overall delivery improvement due to the guaranteed spatio-temporal localization of vascular permeabilization and agent release upon exposure to ultrasound. However, these agents can have a reduced loading capacity and, because new formulations represent unique therapeutic entities, they will require more toxicity and efficacy studies before being approved for clinical use 61. Given the field's proclivity for functionalization, there exists a plethora of conjugated microbubble formulations, detailed elsewhere 61,68,99. The ultrasound-assisted delivery platform has one of the highest potentials for benefit due to the tunability of acoustic parameters and its ability to access deep-seated tumors. Its widespread application requires improvements to both agent formulation and sonication procedures to generate consistent protocols for delivery enhancement.

#### 2.2.3 Hyperthermia

Through local application of heat in a specific temperature range to tumor sites, vascular physiology can be modified as a means of improving drug delivery. While temperatures above 45ºC are associated with tissue ablation and vascular shutdown, sub-ablative temperatures between 39 and 42ºC can help promote vasodilation and increase permeability in tumor vasculature 100,101. This sub-ablative regime, referred to as mild hyperthermia, can be utilized to improve drug extravasation. Among the EPR-adaptive delivery techniques described, hyperthermia is unique in that it is often a side-effect of other external stimuli, including radiation and ultrasound. Alternatively, it can be applied independently through application of external heating, radiofrequency ablation, high-intensity focused ultrasound, or *in vivo* heat production through irradiation of pre-injected photothermal agents 43. Thus, the delivery benefits incurred through mild hyperthermia can be achieved through multiple routes and are of broad importance.

Seminal work by Kong *et al.* compared normothermia (34ºC) to mild hyperthermia (42ºC) for improving extravasation of fluorescent liposomes of various sizes in SKOV-3 ovarian carcinoma xenografts using a window chamber model. They showed that the size range of extravasating agents extended from 7-100 nm to 7-400 nm, as well as observing overall increases in accumulation for all sizes tested. The magnitude of the effect was greatest for smaller nanoparticles and was limited to tumor vasculature 102. Comparable hyperthermic treatment of CAPAN-1 pancreatic xenografts with co-administered liposomal gemcitabine showed a 2-fold reduction in tumor growth when administered biweekly, allowing for a 3-fold reduction in chemotherapy dosing for an equivalent effect 103. They also showed that such an improvement was not achieved using free gemcitabine, highlighting its strength for improving the efficacy of size-selected nanomedicines.

While the extent of improvement appears to be dependent on tumor model, hyperthermia has been shown to work in both EPR-sensitive and insensitive phenotypes. Li *et al.* saw a doubling of agent permeability in murine melanoma and lung models compared to murine sarcoma and human melanoma models 104. They also showed that, while improvements could be maximized by shortening the interval between hyperthermic treatment and delivery, they were still attainable up to 8 hours after pre-treatment, making such a regimen more clinically flexible than co-administration protocols. Lammers *et al.* compared the effects of hyperthermia and radiation to improve accumulation of HPMA co-polymers in both aggressive and slow-growing variants of Dunning R-3327 prostate cancer. They observed that hyperthermia improved uptake of 65kdA poly(HPMA) in the slow-growing model to a level that matched uptake in the aggressive model. However, while radiation treatment was found to increase uptake across all sizes and tumor types, the hyperthermic effect was not recapitulated when tested with smaller, doxorubicin-loaded polymers, suggesting that the result is limited to nanoparticles larger than a certain size threshold 105. This supports the usage of EPR-adaptive delivery strategies to normalize EPR-sensitivity across phenotypes, providing a delivery platform that works reliably in a heterogeneous patient population.

More recently, Bagley *et al.* showed that the increased nanoparticle accumulation seen after hyperthermia treatment was partially regulated by the heat shock response. Their results showed that the increase in accumulation of fluorescent PEGylated iron oxide nanoparticles was hampered after progressive hyperthermia treatments, with no improvement for injections delivered after the third successive hyperthermia treatment. However, when tested in mice with genetically disabled HSF1, a heat shock protein, consistent accumulation dynamics were observed for progressive treatments 106. This suggests that the physiological response to repeated hyperthermia treatment can paradoxically impair future attempts for delivery enhancement, meaning that proper care should be taken when determining treatment cycles for hyperthermia-enabled nano-chemotherapy.

Temperature-sensitive liposomes are one class of nanotherapeutics that capitalizes on these principles. The delivery benefits of the physiological effects of hyperthermia are augmented using liposomes designed to release their contents upon a non-ablative increase in temperature. While it was first explored in the 1970's 107, work by Needham and Dewhirst on ThermoDox® revolutionized the field, including optimizing liposomal formulation 108,109,110, evaluating the release kinetics *in vivo* 111, and testing across a variety of tumor types 112,113. Thermodox® and radiofrequency ablation as a hyperthermia source have been used successfully in early stage clinical trials for the treatment of both non-resectable HCC ((NCT02112656 114) and recurrent chest wall disease in breast cancer patients (NCT00826085 115), with ongoing global phase III trials for HCC treatment currently in progress (NCT02112656 116). While the upper limits of delivery improvement based solely upon physiological changes appears to be lower for hyperthermia than the other delivery platforms, this combination with temperature-sensitive formulations makes it one of the most clinically-advanced strategies discussed. Furthermore, its ease of application and the universal relevance of the vascular heat shock response make hyperthermia-mediated delivery a critical topic of further research.

#### 2.2.4 Photodynamic Therapy

Photodynamic therapy (PDT) has been largely studied in the context of cancer therapy as a means of non-invasively and selectively treating superficial tumor targets. It operates on the principle of stimulating a light-activatable agent (photosensitizer) with NIR light such that, either through direct electron transfer to a biological substrate (type I photosensitization) or to molecular oxygen (type II photosensitization), cytotoxic molecular species are generated. Because the photosensitizer is generally non-toxic in the absence of light and light doses are chosen to be low enough to spare surrounding tissues, the required co-registration of light and sensitizer ensures high localization of cellular and tissue damage to the area of the field of illumination that contains the photosensitizer. Because the penetration depth of NIR light is on the order of millimetres in tissue, this limits application to superficial sites or those that can be probed with cylindrically diffusing optical fibres, such as the prostate 117.

The ability of PDT to cause transient vascular leakiness followed by vessel shutdown has been known since the 1980's, and it was broadly considered to be a detriment to its usage as an adjunctive therapy 118,119,120,121. Snyder *et al.* was one of the first to explore whether or not this mechanism could be utilized to improve agent delivery. In 2003, they illustrated that the application of low fluence phototherapy with a porphyrin-based photosensitizer led to 5-12-fold increase in uptake of fluorescent microspheres ranging in size from 0.1-2 µm but impermeability to microspheres at a smaller size of 0.02 µm. This was complemented with therapeutic studies using liposomal doxorubicin, which showed enhanced accumulation at all doses and improved survival for doses of 3 mg/kg or greater 122. This was soon followed by work from Chen *et al.*, who saw increased uptake of 100 nm FITC-dextran in both subcutaneous and orthotopic murine models of prostate cancer when given 15 minutes after pre-treatment with verteporfin-based PDT 123. Similar findings were observed by Cheng *et al*. with liporubicin delivery to Visudyne-based PDT-treated sarcomas xenografted into rodent lungs, showing both a two-fold increase in accumulation and no change in adjacently treated healthy lung tissue 124.

**Figure 3:**
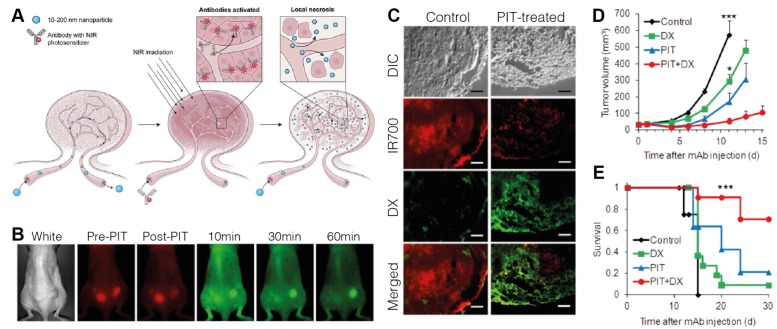
A. Concept behind the use of tumor pre-treatment with antibody-targeted photodynamic therapy (PIT) for improving nanoparticle delivery beyond the EPR effect. B. Fluorescence monitoring of Qdot800 accumulation in bilateral A431 subcutaneous xenografts administered one hour following right-flank treatment with Pan-IR700-mediated PIT. IR700: red, Qdot800: green. C. Histological analysis of intratumoral distribution of IR700 and liposomal daunorubicin (DX) one hour after drug administration, showing that PIT treatment increases extravasation distance and overall accumulation compared to traditional EPR-based delivery. D. Tumor growth inhibition was maximized using a combination of PIT pre-treatment and DX administration. E. Kaplan-Meier curves illustrating prolonged survival following combination treatment. Adapted with permission from 126, copyright 2013 American Chemical Society.

Active targeting strategies have also been employed to improve specificity of PDT-based tactics for improving drug delivery. Mitsunaga *et al.* saw that photodynamic therapy of A431 EGFR+ xenografts 24 hours after injection of IR700 conjugated to panitumumab, a monoclonal antibody specific to EGFR, led to tumor shrinkage via damage to selectively-bound pericytes at the tumor site 125. When followed with treatment using liposomal daunorubicin, they observed a 12.3-fold increase in accumulation and improved survival (Figure 3) 126. When tested in tumors with mixed EGFR expression, delivery was found to be well localized to the EGFR+-expressive areas of the tumor, but still produced an overall delivery improvement of 5-fold with a reduction in tumor burden and prolonged survival, suggesting both photosensitizer selectivity and its activity in tumors of mixed expression 127. Utilizing RGD-modified ferritin as a photosensitizer, Zhen *et al.* also showed the delivery benefits of using active targeting, observing a 20.08-fold increase in accumulation of quantum dots in PDT-treated tumors in a bilateral 4T1 breast tumor xenograft model. They also highlighted a pronounced effect for larger nanoparticles, hypothesizing that the improvement is magnified for agents with size-limited extravasation rates. A lower but apparent effect was still observed for smaller nanoparticles, evidenced by albumin showing increased uptake in breast (4T1, 2.96-fold), prostate (PC-3, 3.39-fold), melanoma (MDA-MB-4355, 2.27-fold), and glioblastoma (U87MG, 5.79-fold) xenografts 128. The potential for specificity of targeted photosensitization is likely highest between all of the discussed physical modalities, making PDT pre-treatment an attractive option for superficial cancer targets.

Overall, the physical EPR-adaptive delivery strategies described here have all been shown to improve nanomedicine uptake and treatment outcomes in preclinical models. While they still require optimization and validation in more clinically relevant models, their basis on mechanisms that can alter tumor structures regardless of the target tumor's level of EPR-sensitivity gives them a greater chance of achieving broad clinical translatability.

## 3. Demand of Preclinical Validation Tools for EPR-adaptive Delivery

**Figure 4:**
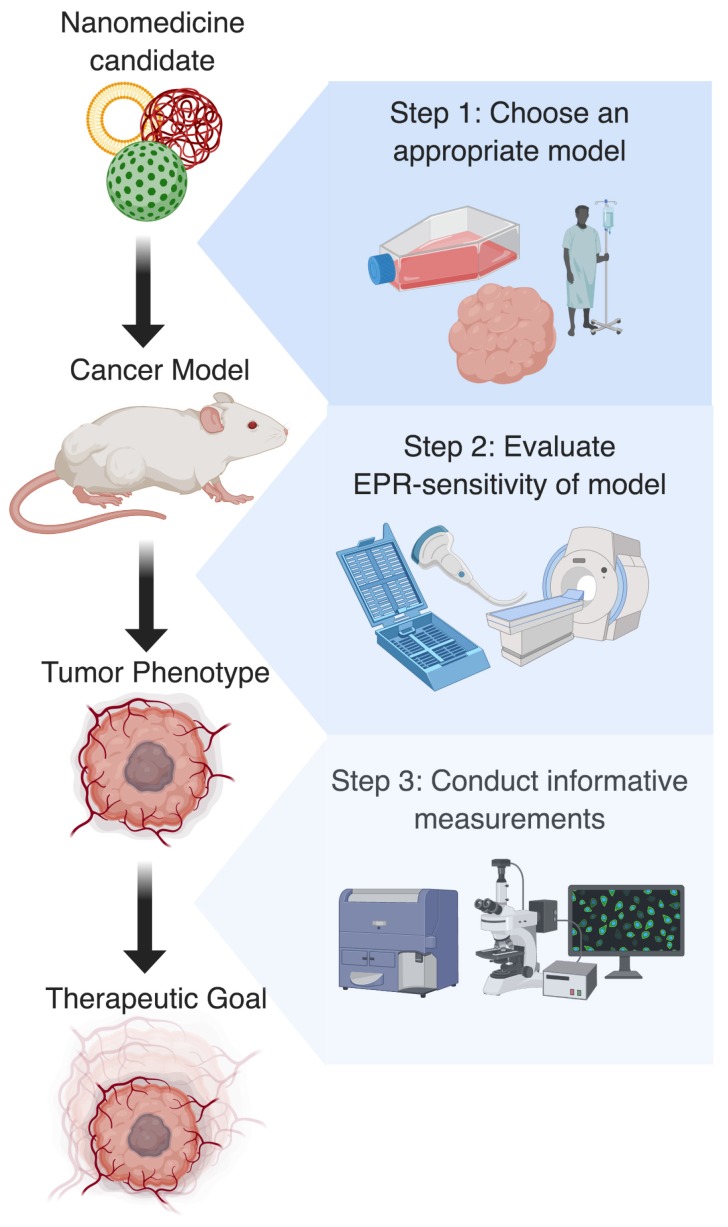
Ideal preclinical workflow for research using EPR-adaptive delivery strategies. Step 1: Based on the clinical target and nanomedicine formulation, the most appropriate tumor model should be identified. Step 2: Following model development, the EPR-sensitivity of the tumor should be assessed using imaging or histology, as this is useful for predicting the magnitude of the expected delivery improvement. Step 3: Finally, a procedure for measuring uptake or therapeutic benefit should be chosen that highlights the spatial or temporal improvement conferred by the delivery strategy. Together, these actions should create more interpretable and generalizable results that will aid in the clinical translation of discoveries.

Whether intentional or through sheer convenience, there is no denying that the current testing paradigm for new innovations in nanomedicine-characterization of a new nanoparticle, assessment of its gross tumor uptake in a xenograft model, and assessment of reduction in tumor growth across a relatively short timeline - is biased towards traditional EPR-based delivery. From the methods used to gauge efficacy to the disease models in which they are validated, there exist numerous implicit advantages which increase the likelihood of observing benefit in any agent that capitalizes on a highly expressed EPR effect. While this has generated excitement with regards to the volume of agents that have shown preclinical promise, its effect on stunting clinical translation has ultimately weakened the field as a whole, giving the impression to many that nanomedicine translation stops at the mouse 129. In addition to over-emphasizing the impact of traditional EPR-based strategies, such a system under-values the benefits of EPR-adaptive delivery strategies. In order to more effectively characterize EPR-adaptive strategies for clinical improvement, a critical reflection on current techniques and an honest confession of what is required must be had (Figure 4).

### 3.1 Assessment of EPR-sensitivity of cancer models

The primary shortcoming of EPR-based nanomedicine delivery is its heterogeneous response in clinical tumors, an outcome which is thought to be the result of inconsistent traits in clinical tumors compared to the common EPR-sensitive phenotype of most preclinical models. In order to both better determine the applicability of a formulation and the eligibility of patients for various treatment options, a reliable means to assess the EPR-sensitivity of tumors is of critical importance.

The classification of tumor phenotypes on the basis of EPR-sensitivity necessitates specific criteria that can be easily assessed, the choice of which are still in early stages of research. This is in part due to a lack of knowledge as to what exactly enables the EPR effect on a mechanistic level. Even if this was discovered, it would still be required that these qualities were valid and measurable at the clinical level. The current techniques characterizing the EPR effect rely mainly on histology and imaging, the latter of which is most attractive due to its non-invasiveness.

Indirect imaging of tumor vasculature is an attractive prospective method for evaluating EPR-sensitivity, as it would allow for a non-invasive, versatile implementation that fits cleanly into established clinical workflows. It is predicated upon first connecting characteristics of tumor vessels or perfusion with agent uptake and assuming that this correlates with improved treatment response, hence its notation as indirect. The Lammers group has made considerable strides in this area using CT 130 and ceUS 14. Correlations between reduced vessel branching as measured by micro-CT and angiogenic potential as assessed by CD31 and SMA immunohistochemistry in a variety of tumor models were discovered, providing one potential metric of vessel quality 130. Furthermore, they found that accumulation of pHPMA-Dy750 in CT26 colon carcinoma xenografts (via FRI) correlated with relative blood volume (via ceUS), illuminating the hypothesized relationship between degree of blood flow and accumulation 14. Sulheim* et al.* combined ceUS, micro-CT, and MRI as tumor imaging modalities to assess vascular characteristics that enable increased uptake of 100 nm fluorospheres in five different animal tumor models. They observed that blood vessel density (via CT) and wash-in time (via ceUS) correlated with improved uptake, while K^trans^, an MRI parameter often used to represent degree of extravasation and vessel leakiness, showed no correlation 15. However, comparable work by Karageorgis *et al.* in a variety of xenograft and orthotopic tumor types using MRI and fluorescence imaging to observe lipo-nanocapsule delivery was not able to find any significant relationship between tumor permeability, vessel size, and blood volume fraction that functioned predictively in their model systems 16.

While these works individually suggest some relationship between vascular features quantifiable on imaging and tumor characteristics representative of EPR-sensitivity, no group has yet found an individual measure that has been shown to be predictive. A unified metric combining multiple features seen on imaging and weighted by degree of correlation may eventually be a useful tool for the prediction of EPR-sensitive phenotypes, but considerable work would be required to validate such a tool across a variety of preclinical and clinical tumor types.

A direct measure of EPR-sensitivity would seek to correlate increased agent uptake with improved efficacy. While this may lead to a more powerful predictive tool for determining tumor phenotype, it also requires additional work to develop trackable components for each new therapeutic to be tested, likely in the form of a companion nanodiagnostic or an incorporable chelator for radiolabeling 39,131. Miller *et al.* showed that FMX, a magnetic nanoparticle used as an MRI contrast agent, could effectively predict nanoparticle accumulation in subcutaneous HT1080 human fibrosarcoma xenografts using PLGA-PEG NPs and therapeutic response using liposomal paclitaxel in subcutaneous 4T1 breast cancer xenografts, respectively. Subjects stratified by high, medium, or low uptake based upon an increase in T2-weighted MRI signal between 1 and 24 hours showed strong correlations with differences in DNA damage, apoptotic fraction, drug uptake, and percent change volume in tumor size 132. Pérez-Medina *et al.* showed similar capabilities with PET imaging, using a liposomal ^89^Zr-nanoreporter and liposomal doxorubicin in mice bearing subcutaneous 4T1 breast cancer xenografts. They illustrated very strong correlations between intensity of PET images at the tumor site and agent accumulation (r=0.97) and were able to provide predictions for therapeutic outcome by stratifying mice into high and low accumulation subgroups (threshold of 25 mg/kg interpolated uptake at the tumor site), displaying categorical differences in relative tumor size increase and median survival 133. Both of these studies illustrate the power of these techniques in preclinical models that display reasonably high levels of uptake heterogeneity, giving promise to future clinical application of these techniques.

The clinical viability of companion nanodiagnostics has been trialed by Merrimack Pharmaceuticals in two studies in human patients. The first study related therapeutic response from liposomal irontecan with accumulation of FMX in 28 patients with advanced, metastatic cancer. While they observed only marginal correlation between irontecan uptake and FMX signal, they did see a relationship between reduction in lesion size and FMX signal after stratifying patients by median degree of FMX accumulation 18. The second study treated nineteen HER2+ metastatic breast cancer patients with ^64^Cu-labelled HER2-targeted PEGylated liposomal doxorubicin and transtuzumab, monitoring biodistribution using PET/CT scans and biopsies for comparison with therapeutic response. Lesion volume was not found to correlate with agent uptake, and lesion deposition was found to vary between 0.5 and 14% ID/g. When stratified by lowest uptake lesion, agent accumulation was found to significantly correlate with therapeutic response, giving clinical evidence that EPR-sensitivity is a determinant of nanomedicine efficacy 17. While both of these studies provide a clinical basis for EPR-sensitivity, it should be noted that the unintuitive manner by which their statistical groupings were performed merits additional work to validate whether or not the predictive power of these groupings extends to a broader clinical population. Future work conducted in patients before receiving radiation and chemotherapy would also be key to deducing the true strength of the relationship between uptake and therapeutic benefit, including whether or not it can be used as a metric for EPR-sensitivity.

The combined use of indirect and direct methods for evaluating EPR-sensitivity, while still early in development, poses significant benefit to the developmental paradigm for nanomedicine. For existing formulations, it would provide a means by which patients can be pre-selected on the basis of their EPR-sensitivity and thereby potential to see a positive therapeutic response. For new platforms, it would be an invaluable preclinical validator for studies that want to investigate if agent accumulation and treatment response in EPR-insensitive phenotypes can be made comparable to EPR-sensitive phenotypes through the use of EPR-adaptive delivery strategies.

### 3.2 Choice of an appropriate preclinical model

There is no doubt that the rapid rate at which new nanomedicines have been developed is partially due to the incredibly convenient and accessible preclinical models available for testing. Primary among these are subcutaneous and orthotopic xenografts, a system almost synonymously used for early proof-of-efficacy studies. While these models possess highly desirable features including rapid growth and relative homogeny between subjects, they exhibit a radically different microenvironment than clinical tumors, whereby the majority of these distinctions make them more amenable to traditional EPR-based nanomedicine delivery. High rates of angiogenesis in xenografts lead to uniformly low pericyte coverage and large endothelial fenestrations 105,130, enhancing extravasation potential 134, while clinical counterparts have more heterogeneity in vessel leakiness, often comprising of both vasculature with complete pericyte coverage and vasculature with total absence of fenestrations 135,136. Additionally, the high tumor-to-body ratio achieved in rodent models also biases towards treatments that capitalize on increased exposure to tumor vessels through extended circulation time. Given that both humans and mice have comparable perfusion dynamics when normalized by weight, conservative estimates show that a 2g tumor in a 20g mouse would experience a 100-fold increase in circulation-mediated exposure to an injected agent compared to a 70g tumor in a 70kg human, giving room for significant error if results regarding efficacy are extrapolated without justification. Moreover, the slow-growing nature of human cancers under immune pressure results in a complex, genetically-diverse microenvironment with a dense extracellular matrix and high interstitial fluid pressure, all of which reduce nanoparticle motility and potential for extravasation compared to fast-growing animal models 2,3,4,136,137,138,139. These are but a selection of a list of features that illustrate the shortcomings of our most prevalent model systems, all of which are knowingly accepted in the name of study throughput.

In addition to exaggerating efficacy of most nanomedicines, these models are poorly suited for testing EPR-adaptive delivery strategies. Given that EPR-adaptive techniques attempt to elevate delivery to cancers with microenvironmental features that prohibit conventional delivery, a more representative test of efficacy would use a comparison between an EPR-sensitive tumor type and a more clinically-relevant EPR-insensitive tumor type. The latter would provide the largest margin for improvement compared to conventional delivery strategies, while the comparison would illustrate generalizable efficacy across large changes in the tumor microenvironment.

Alternative models that may be more clinically representative include patient-derived xenografts (PDX), organoids, and spontaneous cancers, including genetically-engineered models. PDX allow for the transplantation of tumor cells directly from a patient into an animal model, often preserving stromal structures and allowing for testing against specific, low-incidence cancer phenotypes 140. While these models require clinical access and host lower engraftment rates than xenografts, they have shown promising results - work by Zhou *et al.* in an orthotopic pancreatic PDX showed comparable collagen and fibroblast-activated protein levels to the native tumor and, when treated with either IGF1R-targeted IONP-doxorubicin or free doxorubicin, showed increased tumor growth regression and apoptotic cell death in the nanoformulation treatment arm 141.

Organoids, 3D cancer cell cultures that better recapitulate the ECM and stroma when compared to 2D systems, may be useful as a supplementary tool to assess the impact of EPR-adaptive delivery strategies on various characteristics of the tumor microenvironment, including stromal density 142. While the diffusion-dominated dynamics of organoids make them undesirable for assessment of nanoparticle delivery in tumors known to exhibit convection-dominated transport 3,68, their utility as a cellular phantom may enable high throughput studies that are infeasible using more complex *in vivo* models.

Finally, spontaneous cancers are claimed to be one of the tumor models most representative of slow-growing human cancers. They have shown some success, with Cabral *et al.* illustrating that treatment using DACHPt-loaded micelles showed significant benefits for survival, reduced tumor burden, and decreased metastasis when compared to free oxaliplatin in a spontaneous pancreatic cancer model 143. However, they are not infallible: Deshantri *et al.* compared liposomal and free prednisolone in the treatment of spontaneous breast carcinomas, and they observed accumulation of their liposomal formulation at comparable levels to their studies in B16F10 melanoma and C26 colon xenografts 144. Thus, spontaneous cancer models should still be scrutinized based on the phenotypic sensitivity to EPR-based delivery rather than immediately embraced as superior models, although their usage does represent a productive shift towards meaningful cancer model selection.

Each new model systems brings with it its own set of advantages and disadvantages, with no single choice superior to others when assessed cumulatively based on clinical relevance, throughput potential, technical difficulty, and resource investment. Thus, rather than being seen as an indictment of the xenograft, this discussion should be viewed as an argument for a more informed choice of experimental model that best suits the research question being put forward. Diversifying the variety of models in nanomedicine research will not only allow for better options in testing EPR-adaptive therapies, but it will also better reflect the clinical reality of a heterogeneous tumor population, yielding a more fruitful environment for nanomedicine development.

### 3.3 Optimal measurements for detecting delivery and gauging efficacy

The early decades of nanomedicine research equivocated improvements in overall accumulation of nanoparticles within tumors with improvements in delivery, as this was often found to be correlated with therapeutic benefits in the form of decreased tumor burden and prolonged survival. In the wake of our growing appreciation of the complexity of the tumor microenvironment, it is surprising that this relationship is still accepted with little question - a large number of preclinical works claim improved delivery using only gross measurements such as UPLC, fluorescence spectroscopy, mass spectroscopy, and gamma counting of excised and digested tumors. High-resolution imaging has the capacity to provide spatial localization of agents within a tumor, but many studies that quantify the delivery of functionalized or multimodal nanodiagnostics do so by averaging fluorescence, PET, or MRI signal across the entire tumor area. While this is in some cases a technological limitation, there is often little discussion of how gross measurements and averaged signals are not directly representative of improved delivery to the intended, cellular target. Since several of the key advantages conferred by an EPR-adaptive delivery approach are spatial and temporal in nature, the use of appropriate measurements is key in proving their mechanism and illuminating their benefits for delivery.

As spatial measures, penetration depth and cellular localization are two areas where EPR-adaptive delivery may provide significant improvements compared to passive accumulation. It has long been accepted that nanoparticles uptaken by virtue of the EPR effect remain close to blood vessels, leading to the phenomenon of the “rim effect” wherein the hypervascularized tumor periphery exhibits greater accumulation than the less vascularized, more necrotic tumor core 145,146,147. Radiation 46,147, ultrasound 65,66,68, and hyperthermia treatment 104,111 have all been shown to improve extravasation distance from vessels while maintaining or improving overall uptake. Intravital confocal or two-photon microscopy has proven to be a strong, *in vivo* tool for evaluating extravasation distance over time, although it often necessitates the use a window-chamber model. Immunohistochemistry of tumor sections can also help to correlate protein expression and intratumoral distribution, although it becomes less suitable if extravasation measurements at multiple timepoints are desired. Cellular localization of delivered nanomedicine is another measure that is more informative of therapeutic outcome than gross tumor accumulation. From *in vitro* studies, ultrasound-assisted delivery is known to induce cellular localization through several mechanisms 148,149,150,151 and PDT can facilitate cytosolic delivery through photochemical internalization if photosensitive agents are co-loaded with therapeutic agents, leading to endolysosomal damage 152. Flow cytometry is used in this *in vitro* work, but few researchers have made the needed transition to re-validating these findings for *in vivo* studies. This is especially pertinent to experiments performed in immunocompetent models, as immune cells like tumor-associated macrophages are known to uptake a significant proportion of accumulated nanoparticles 43,132,153.

Temporal resolution of nanoparticle uptake is another aspect of the delivery paradigm that is rarely questioned. Many studies that rely upon end-point metrics of nanoparticle accumulation, such as histology, UPLC, and spectroscopy, choose a single timepoint for measuring accumulation. This timepoint is often chosen to be 24 hours or later, given the time dependence of the EPR effect for progressive deposition up to 72 hours after injection 1,154. However, growing evidence suggests more complex uptake dynamics, including size-dependent nanoparticle uptake and clearance occurring over the first 24 hours 155. While these differences at short timescales tend to equalize at later timepoints in xenografts 13, a transition to more clinically-representative tumor models may not show the same degree of convergence. For instance, one ultrasound-assisted drug delivery method discovered by Huynh *et al.* utilizes an *in vivo* conversion of microbubbles to stable nanoparticles for enhancing agent loading fraction. They showcased peak intact nanoparticle accumulation as early as 15 minutes after ultrasound exposure, exhibiting an atypical mechanism of uptake (Figure 2G) 156. The use of multimodal nanoparticles or companion nanodiagnostics for progressive imaging after treatment can provide qualitative evidence towards the rate of accumulation, but techniques which can truly combine both spatial and temporal specificity, such as intravital microscopy, micro-CT, or ceUS, will be the gold standard moving forward for evaluating EPR-adaptive delivery strategies.

Finally, computational modeling may also be useful as a companion tool for testing EPR-adaptive delivery. Models that incorporate research data to help better describe an observed effect can be useful for both hypothesis generation and elucidation of mechanism. For instance, Snyder *et al.* used a variant of the Hill concentration-effect equation alongside data from PDT-treated and untreated tumors to better evaluate whether or not their observed therapeutic benefits were due to additive or synergistic effects of their combination treatment 122. This helps generate a larger understanding of the impact of more complicated, multi-step treatment strategies. As another tool, Arvanitis *et al.* produced a data-driven mathematical model to simulate the effects of focused ultrasound treatment on diffusive and convective transport out of tumor vasculature and through the tumor microenvironment, helping them interpret their observations. Similarly, Miller *et al.*'s modelling of uptake of nanoparticles and companion nanodiagnostics allowed them to hypothesize tumor characteristics that primarily drive uptake for different particle types 132. When informed by relevant data parameters, modeling has the potential to extend important research findings and help researcher's develop a more complete understanding of drug delivery dynamics in complex tumors.

## 4. The Strength of Generalizability: Moving Forward

While the EPR effect has been an incredible motivating force behind decades of nanomedicine development, the era of embracing it as an infallible solution has passed. Three decades of nanomedicine research has produced only a handful of useful clinical formulations - a far cry away from the “magic bullet” as it had been marketed. While this does not compromise its validity, it defines the scope of its function; namely, its primary efficacy against EPR-sensitive cancer phenotypes rather than the vast heterogeneity of all clinical cancers for which it has been purported to benefit. To broaden the applicability of past decades of research, EPR-adaptive delivery strategies are necessary to deliver agents in a manner suitable for a larger selection of tumor types. Generalizability is one of the strongest aspects of this emerging delivery paradigm, and it seeks to bridge the long-lasting issue of clinical translatability in the field of nanomedicine.

In order to aid in the development of these techniques, the field needs to critically reflect on the biases inherent to the current methodologies we use in preclinical research, and a conscious decision must be made to use more representative models and systems. To achieve this larger gain for the community, it may be necessary for journals to use firmer criteria with regards to evaluation of preclinical studies, requiring stipulations on how researchers frame their results based on the limitations of the models, measurements, and techniques that they have used.

Because EPR-adaptive delivery techniques do not rely upon the tumor-specific EPR effect, it also enables their translation to other clinical targets. As nanoparticles can be extensively functionalized to provide contrast for almost all clinically available imaging modalities, a tool that improves their access to cells and structures beyond the vasculature would be invaluable to the diagnosis of disease across almost all organ systems. There is potential for circulation-privileged areas such as the brain and spinal cord to be accessed and treated non-invasively. Immunotherapy applications would benefit from functionalized T-cell delivery to specific organ systems, a task that sees natural benefit from these localized delivery platforms. A shift towards a non-invasive delivery archetype that is highly localized benefits the whole genre of design-based clinical research - it is time we start giving it the attention that it deserves.

## Figures and Tables

**Table 1:** Summary of physical strategies for EPR-adaptive delivery

Technique	Physiological Effect	Strengths	Weaknesses
**Radiation**	Decreased intratumoral and interstitial fluid pressure, reduced perfusion, alteration of ECM and vessel growth	Established therapeutic benefit, fits clinical workflow, high penetration depth, utilizes existing clinical resources	Radiation dose and fractionation schedule must be optimized for different tumor types to prevent delivery impairment, damage to surrounding tissue
**Ultrasound**	Transient disruption of endothelium increases vascular permeability	High penetration depth, non-invasive, localized, minimal damage to surrounding tissue, amenable to repeated treatments, can use existing clinically-approved microbubbles	Some strategies require image guidance, some techniques are not compatible with current clinical ultrasound systems
**Hyperthermia**	Vasodilation, increased vessel permeability	Versatile modes of delivery, potentially non-invasive, localized, exploitable side-effect of other external stimuli	Delivery resistance after repeat sessions, size limitations on eligible sensitizing agents
**Photodynamic Therapy**	Damage to vessels causes transient vascular leakiness	Co-registration of photosensitizer and applied light gives high specificity to area of illumination, potentially non-invasive	Low penetration depth of light necessitates superficial targets or invasive light delivery probes, delay required for photosensitizer build-up before light administration extends clinical burden

## Abbreviations

EPR: enhanced permeability and retention
PEG: polyethylene glycol
IFP: interstitial fluid pressure
ID/g: injected dose per gram
ECM: extraceullar matrix
PX-TXL: poly(L-glutamic)-acid-conjugated paclitaxel
MRI: magnetic resonance imaging
PET: positron emission tomography
HPMA: *N*-(2-hydroxypropyl) methacrylamide
HCC: hepatocellular carcinoma
PDT: photodynamic therapy
NIR: near-infrared
FITC: fluorescein
EGFR: epidermal growth factor receptor
RGD: arginine-glycine-aspartate
FRI: florescence reflectance imaging
CT: computed tomography
ceUS: contrast-enhanced ultrasound
α-SMA: alpha-smooth muscle actin
FMX: ferumoxytol
HER2: human epidermal growth factor 2
PDX: patient-derived xenograft
IONP: iron oxide nanoparticle
DACHPt: Dichloro(1,2-Diaminocyclohexane)Platinum(II)
UPLC: ultra-performance liquid chromatography

## References

[1] Matsumura Y, Maeda H (1986). A new concept for macromolecular therapeutics in cancer chemotherapy: mechanism of tumoritropic accumulation of proteins and the antitumor agent smancs. Cancer Res.

[2] Jain RK (1987). Transport of molecules in the tumor interstitium: a review. Cancer Res.

[3] Boucher Y, Baxter LT, Jain RK (1990). Interstitial pressure gradients in tissue-isolated and subcutaneous tumors: implications for therapy. Cancer Res.

[4] Nichols JW, Bae YH (2014). EPR: Evidence and fallacy. J Control Release.

[5] Gabizon A, Shmeeda H, Barenholz Y (2003). Pharmacokinetics of Pegylated Liposomal Doxorubicin. Clin Pharmacokinet.

[6] O'Brien MER, Wigler N, Inbar M (2004). Reduced cardiotoxicity and comparable efficacy in a phase III trial of pegylated liposomal doxorubicin HCl (CAELYX^TM^/Doxil®) versus conventional doxorubicin for first-line treatment of metastatic breast cancer. Ann Oncol.

[7] Prabhakar U, Maeda H, Jain RK (2013). Challenges and key considerations of the enhanced permeability and retention effect for nanomedicine drug delivery in oncology. Cancer Res.

[8] Maeda H (2015). Toward a full understanding of the EPR effect in primary and metastatic tumors as well as issues related to its heterogeneity. Adv Drug Deliv Rev.

[9] Harrington KJ, Rowlinson-Busza G, Syrigos KN (2001). Effective Targeting of Solid Tumors in Patients With Locally Advanced Cancers by Radiolabeled Pegylated Liposomes. Clin Cancer Res.

[10] Hansen AE, Petersen AL, Henriksen JR (2015). Positron Emission Tomography Based Elucidation of the Enhanced Permeability and Retention Effect in Dogs with Cancer Using Copper-64 Liposomes. ACS Nano.

[11] Danhier F, Feron O, Préat V (2010). To exploit the tumor microenvironment: Passive and active tumor targeting of nanocarriers for anti-cancer drug delivery. J Control Release.

[12] Mura S, Nicolas J, Couvreur P (2013). Stimuli-responsive nanocarriers for drug delivery. Nat Mater.

[13] Heneweer C, Holland JP, Divilov V, Carlin S, Lewis JS (2011). Magnitude of Enhanced Permeability and Retention Effect in Tumors with Different Phenotypes: 89Zr-Albumin as a Model System. J Nucl Med.

[14] Theek B, Gremse F, Kunjachan S (2014). Characterizing EPR-mediated passive drug targeting using contrast-enhanced functional ultrasound imaging. J Control Release.

[15] Sulheim E, Kim J, van Wamel A (2018). Multi-modal characterization of vasculature and nanoparticle accumulation in five tumor xenograft models. J Control Release.

[16] Karageorgis A, Dufort S, Sancey L (2016). An MRI-based classification scheme to predict passive access of 5 to 50-nm large nanoparticles to tumors. Sci Rep.

[17] Lee H, Shields AF, Siegel BA (2017). 64Cu-MM-302 Positron Emission Tomography Quantifies Variability of Enhanced Permeability and Retention of Nanoparticles in Relation to Treatment Response in Patients with Metastatic Breast Cancer. Clin Cancer Res.

[18] Ramanathan RK, Korn RL, Raghunand N (2017). Correlation between Ferumoxytol Uptake in Tumor Lesions by MRI and Response to Nanoliposomal Irinotecan in Patients with Advanced Solid Tumors: A Pilot Study. Clin Cancer Res.

[19] Danhier F (2016). To exploit the tumor microenvironment: Since the EPR effect fails in the clinic, what is the future of nanomedicine?. J Control Release.

[20] Maeda H, Nakamura H, Fang J (2013). The EPR effect for macromolecular drug delivery to solid tumors: Improvement of tumor uptake, lowering of systemic toxicity, and distinct tumor imaging in vivo. Adv Drug Deliv Rev.

[21] Reichman HR, Farrell CL, Del Maestro RF (1986). Effects of steroids and nonsteroid anti-inflammatory agents on vascular permeability in a rat glioma model. J Neurosurg.

[22] Maeda H (2017). Polymer therapeutics and the EPR effect. J Drug Target.

[23] Islam W, Fang J, Imamura T (2018). Augmentation of the Enhanced Permeability and Retention Effect with Nitric Oxide-Generating Agents Improves the Therapeutic Effects of Nanomedicines. Mol Cancer Ther.

[24] Wu J, Akaike T, Maeda H (1998). Modulation of enhanced vascular permeability in tumors by a bradykinin antagonist, a cyclooxygenase inhibitor, and a nitric oxide scavenger. Cancer Res.

[25] Nagamitsu A, Greish K, Maeda H (2009). Elevating Blood Pressure as a Strategy to Increase Tumor-targeted Delivery of Macromolecular Drug SMANCS: Cases of Advanced Solid Tumors. Jpn J Clin Oncol.

[26] Seki T, Fang J, Maeda H (2009). Enhanced delivery of macromolecular antitumor drugs to tumors by nitroglycerin application. Cancer Sci.

[27] Jain RK (2001). Normalizing tumor vasculature with anti-angiogenic therapy: A new paradigm for combination therapy. Nat Med.

[28] Chen Q, Xu L, Chen J (2017). Tumor vasculature normalization by orally fed erlotinib to modulate the tumor microenvironment for enhanced cancer nanomedicine and immunotherapy. Biomaterials.

[29] Choi J, Credit K, Henderson K (2006). Intraperitoneal Immunotherapy for Metastatic Ovarian Carcinoma: Resistance of Intratumoral Collagen to Antibody Penetration. Clin Cancer Res.

[30] Eikenes L, Tari M, Tufto I, Bruland ØS, de Lange Davies C (2005). Hyaluronidase induces a transcapillary pressure gradient and improves the distribution and uptake of liposomal doxorubicin (Caelyx^TM^) in human osteosarcoma xenografts. Br J Cancer.

[31] Wang H, Han X, Dong Z (1902). Hyaluronidase with pH-responsive Dextran Modification as an Adjuvant Nanomedicine for Enhanced Photodynamic-Immunotherapy of Cancer. Adv Funct Mater.

[32] Gong H, Chao Y, Xiang J (2016). Hyaluronidase To Enhance Nanoparticle-Based Photodynamic Tumor Therapy. Nano Lett.

[33] Michl P, Gress TM (2012). Improving drug delivery to pancreatic cancer: breaching the stromal fortress by targeting hyaluronic acid. Gut.

[34] Wang J, Lu Z, Gao Y, Wientjes MG, Au JL-S (2011). Improving delivery and efficacy of nanomedicines in solid tumors: role of tumor priming. Nanomedicine.

[35] Jang SH, Wientjes MG, Au JL-S (2001). Enhancement of Paclitaxel Delivery to Solid Tumors by Apoptosis-Inducing Pretreatment: Effect of Treatment Schedule.

[36] Chauhan VP, Martin JD, Liu H (2013). Angiotensin inhibition enhances drug delivery and potentiates chemotherapy by decompressing tumour blood vessels. Nat Commun.

[37] Diop-Frimpong B, Chauhan VP, Krane S, Boucher Y, Jain RK (2011). Losartan inhibits collagen I synthesis and improves the distribution and efficacy of nanotherapeutics in tumors. Proc Natl Acad Sci.

[38] Murphy JE, Wo JY, Ryan DP (2019). Total Neoadjuvant Therapy With FOLFIRINOX in Combination With Losartan Followed by Chemoradiotherapy for Locally Advanced Pancreatic Cancer.

[39] Golombek SK, May J-N, Theek B (2018). Tumor targeting via EPR: Strategies to enhance patient responses. Adv Drug Deliv Rev.

[40] Nakamura Y, Mochida A, Choyke PL, Kobayashi H (2016). Nanodrug Delivery: Is the Enhanced Permeability and Retention Effect Sufficient for Curing Cancer?. Bioconjug Chem.

[41] Maeda H, Fang J, Inutsuka T, Kitamoto Y (2003). Vascular permeability enhancement in solid tumor: various factors, mechanisms involved and its implications. Int Immunopharmacol.

[42] Fang J, Nakamura H, Maeda H (2011). The EPR effect: Unique features of tumor blood vessels for drug delivery, factors involved, and limitations and augmentation of the effect. Adv Drug Deliv Rev.

[43] Overchuk M, Zheng G (2018). Overcoming obstacles in the tumor microenvironment: Recent advancements in nanoparticle delivery for cancer theranostics. Biomaterials.

[44] Stapleton S, Jaffray D, Milosevic M (2017). Radiation effects on the tumor microenvironment: Implications for nanomedicine delivery. Adv Drug Deliv Rev.

[45] Li C, Ke S, Wu Q-P (2000). Tumor Irradiation Enhances the Tumor-specific Distribution of Poly(L-glutamic acid)-conjugated Paclitaxel and Its Antitumor Efficacy. Clin Cancer Res.

[46] Davies C de L, Lundstrøm LM, Frengen J (2004). Radiation Improves the Distribution and Uptake of Liposomal Doxorubicin (Caelyx) in Human Osteosarcoma Xenografts. Cancer Res.

[47] Koukourakis MI, Koukouraki S, Giatromanolaki A (1999). Liposomal doxorubicin and conventionally fractionated radiotherapy in the treatment of locally advanced non-small-cell lung cancer and head and neck cancer. J Clin Oncol.

[48] Varveris H, Kachris S, Mazonakis M (2004). Pegulated liposomal doxorubicin and cisplatin given concurrently with conventional radiotherapy: A phase I dose-escalation trial for patients with squamous cell carcinoma of head and neck and lung. Oncol Rep.

[49] Koukourakis MI, Patlakas G, Froudarakis ME (2007). Hypofractionated accelerated radiochemotherapy with cytoprotection (Chemo-HypoARC) for inoperable non-small cell lung carcinoma. Anticancer Res.

[50] Tsoutsou PG, Froudarakis ME, Bouros D, Koukourakis MI (2008). Hypofractionated/accelerated radiotherapy with cytoprotection (HypoARC) combined with vinorelbine and liposomal doxorubicin for locally advanced non-small cell lung cancer (NSCLC). Anticancer Res.

[51] Rosenthal DI, Yom SS, Liu L (2002). A Phase I Study of SPI-077 (Stealth® Liposomal Cisplatin) Concurrent with Radiation Therapy for Locally Advanced Head and Neck Cancer. Invest New Drugs.

[52] Koukourakis MI, Manavis J, Simopoulos C (2005). Hypofractionated accelerated radiotherapy with cytoprotection combined with trastuzumab, liposomal doxorubicine, and docetaxel in c-erbB-2-positive breast cancer. Am J Clin Oncol.

[53] Koukourakis Sofia Koukouraki, Alexa MI (2000). High Intratumoral Accumulation of Stealth Liposomal Doxorubicin in Sarcomas: Rationale for Combination with Radiotherapy. Acta Oncol (Madr).

[54] Calais G, Alfonsi M, Bardet E (1999). Randomized Trial of Radiation Therapy Versus Concomitant Chemotherapy and Radiation Therapy for Advanced-Stage Oropharynx Carcinoma. JNCI J Natl Cancer Inst.

[55] Machtay M, Moughan J, Trotti A (2008). Factors associated with severe late toxicity after concurrent chemoradiation for locally advanced head and neck cancer: an RTOG analysis. J Clin Oncol.

[56] Znati CA, Rosenstein M, Boucher Y (1996). Effect of radiation on interstitial fluid pressure and oxygenation in a human tumor xenograft. Cancer Res.

[57] Barker HE, Paget JTE, Khan AA, Harrington KJ (2015). The tumour microenvironment after radiotherapy: mechanisms of resistance and recurrence. Nat Rev Cancer.

[58] Stapleton S, Milosevic M, Tannock IF, Allen C, Jaffray DA (2015). The intra-tumoral relationship between microcirculation, interstitial fluid pressure and liposome accumulation. J Control Release.

[59] Maeda A, Leung MKK, Conroy L (2012). In Vivo Optical Imaging of Tumor and Microvascular Response to Ionizing Radiation. PLoS One.

[60] Dimcevski G, Kotopoulis S, Bjånes T (2016). A human clinical trial using ultrasound and microbubbles to enhance gemcitabine treatment of inoperable pancreatic cancer. J Control Release.

[61] Lammertink BHA, Bos C, Deckers R (2015). Sonochemotherapy: from bench to bedside. Front Pharmacol.

[62] Goertz DE (2015). An overview of the influence of therapeutic ultrasound exposures on the vasculature: High intensity ultrasound and microbubble-mediated bioeffects. Int J Hyperth.

[63] Kooiman K, Emmer M, Foppen-Harteveld M, van Wamel A, de Jong N (2010). Increasing the Endothelial Layer Permeability Through Ultrasound-Activated Microbubbles. IEEE Trans Biomed Eng.

[64] Juffermans LJM, van Dijk A, Jongenelen CAM (2009). Ultrasound and Microbubble-Induced Intra- and Intercellular Bioeffects in Primary Endothelial Cells. Ultrasound Med Biol.

[65] Wang T-Y, Choe JW, Pu K (2015). Ultrasound-guided delivery of microRNA loaded nanoparticles into cancer. J Control Release.

[66] Theek B, Baues M, Ojha T (2016). Sonoporation enhances liposome accumulation and penetration in tumors with low EPR. J Control Release.

[67] Tardy I, Pochon S, Theraulaz M (2010). Ultrasound Molecular Imaging of VEGFR2 in a Rat Prostate Tumor Model Using BR55.

[68] Arvanitis CD, Askoxylakis V, Guo Y (2018). Mechanisms of enhanced drug delivery in brain metastases with focused ultrasound-induced blood-tumor barrier disruption. Proc Natl Acad Sci.

[69] Hynynen K (2008). Ultrasound for drug and gene delivery to the brain. Adv Drug Deliv Rev.

[70] Choi JJ, Selert K, Vlachos F, Wong A, Konofagou EE (2011). Noninvasive and localized neuronal delivery using short ultrasonic pulses and microbubbles. Proc Natl Acad Sci U S A.

[71] Park J, Zhang Y, Vykhodtseva N, Jolesz FA, McDannold NJ (2012). The kinetics of blood brain barrier permeability and targeted doxorubicin delivery into brain induced by focused ultrasound. J Control Release.

[72] Liu H-L, Fan C-H, Ting C-Y, Yeh C-K (2014). Combining Microbubbles and Ultrasound for Drug Delivery to Brain Tumors: Current Progress and Overview. Theranostics.

[73] Fan C-H, Ting C-Y, Chang Y-C (2015). Drug-loaded bubbles with matched focused ultrasound excitation for concurrent blood-brain barrier opening and brain-tumor drug delivery. Acta Biomater.

[74] Treat LH, McDannold N, Vykhodtseva N (2007). Targeted delivery of doxorubicin to the rat brain at therapeutic levels using MRI-guided focused ultrasound. Int J Cancer.

[75] Treat LH, McDannold N, Zhang Y, Vykhodtseva N, Hynynen K (2012). Improved Anti-Tumor Effect of Liposomal Doxorubicin After Targeted Blood-Brain Barrier Disruption by MRI-Guided Focused Ultrasound in Rat Glioma. Ultrasound Med Biol.

[76] Hynynen K Blood-Brain Barrier Disruption Using Transcranial MRI-Guided Focused Ultrasound. https://clinicaltrials.gov/ct2/show/NCT02343991.

[77] Unga J, Hashida M (2014). Ultrasound induced cancer immunotherapy. Adv Drug Deliv Rev.

[78] Snipstad S, Berg S, Mørch Ý et al (2017). Ultrasound improves the delivery and therapeutic effect of nanoparticle-stabilized microbubbles in breast cancer xenografts. Ultrasound Med Biol.

[79] Lin C-Y, Liu T-M, Chen C-Y (2010). Quantitative and qualitative investigation into the impact of focused ultrasound with microbubbles on the triggered release of nanoparticles from vasculature in mouse tumors. J Control Release.

[80] Lentacker I, De Smedt SC, Sanders NN (2009). Drug loaded microbubble design for ultrasound triggered delivery. Soft Matter.

[81] Klibanov AL (2006). Microbubble Contrast Agents: Targeted Ultrasound Imaging and Ultrasound-Assisted Drug-Delivery Applications. Invest Radiol.

[82] Ibsen S, Schutt CE, Esener S (2013). Microbubble-mediated ultrasound therapy: a review of its potential in cancer treatment. Drug Des Devel Ther.

[83] Klibanov AL, Shevchenko TI, Raju BI, Seip R, Chin CT (2010). Ultrasound-triggered release of materials entrapped in microbubble-liposome constructs: A tool for targeted drug delivery. J Control Release.

[84] Chang S, Guo J, Sun J (2013). Targeted microbubbles for ultrasound mediated gene transfection and apoptosis induction in ovarian cancer cells. Ultrason Sonochem.

[85] Kheirolomoom A, Dayton PA, Lum AFH (2007). Acoustically-active microbubbles conjugated to liposomes: Characterization of a proposed drug delivery vehicle. J Control Release.

[86] Yan F, Li L, Deng Z (2013). Paclitaxel-liposome-microbubble complexes as ultrasound-triggered therapeutic drug delivery carriers. J Control Release.

[87] Liu X, Gong P, Song P (2018). Fast functionalization of ultrasound microbubbles using strain promoted click chemistry. Biomater Sci.

[88] Slagle CJ, Thamm DH, Randall EK, Borden MA (2018). Click Conjugation of Cloaked Peptide Ligands to Microbubbles. Bioconjug Chem.

[89] Geers B, Lentacker I, Sanders NN (2011). Self-assembled liposome-loaded microbubbles: The missing link for safe and efficient ultrasound triggered drug-delivery. J Control Release.

[90] Tinkov S, Coester C, Serba S (2010). New doxorubicin-loaded phospholipid microbubbles for targeted tumor therapy: In-vivo characterization. J Control Release.

[91] Park J Il, Jagadeesan D, Williams R (2010). Microbubbles Loaded with Nanoparticles: A Route to Multiple Imaging Modalities. ACS Nano.

[92] Crake C, Owen J, Smart S (2016). Enhancement and Passive Acoustic Mapping of Cavitation from Fluorescently Tagged Magnetic Resonance-Visible Magnetic Microbubbles In Vivo. Ultrasound Med Biol.

[93] Barrefelt ÅA, Brismar TB, Egri G (2013). Multimodality imaging using SPECT/CT and MRI and ligand functionalized 99mTc-labeled magnetic microbubbles. EJNMMI Res.

[94] Niu C, Wang Z, Lu G (2013). Doxorubicin loaded superparamagnetic PLGA-iron oxide multifunctional microbubbles for dual-mode US/MR imaging and therapy of metastasis in lymph nodes. Biomaterials.

[95] Tartis MS, Kruse DE, Zheng H (2008). Dynamic microPET imaging of ultrasound contrast agents and lipid delivery. J Control Release.

[96] Xu RX, Huang J, Xu JS (2009). Fabrication of indocyanine green encapsulated biodegradable microbubbles for structural and functional imaging of cancer. J Biomed Opt.

[97] Ke H, Xing Z, Zhao B (2009). Quantum-dot-modified microbubbles with bi-mode imaging capabilities. Nanotechnology.

[98] Jin B, Lin M, You M (2015). Microbubble embedded with upconversion nanoparticles as a bimodal contrast agent for fluorescence and ultrasound imaging. Nanotechnology.

[99] Boissenot T, Bordat A, Fattal E, Tsapis N (2016). Ultrasound-triggered drug delivery for cancer treatment using drug delivery systems: From theoretical considerations to practical applications. J Control Release.

[100] Dudar TE, Jain RK (1984). Differential Response of Normal and Tumor Microcirculation to Hyperthermia. Cancer Res.

[101] Burd R, Dziedzic TS, Xu Y (1998). Tumor cell apoptosis, lymphocyte recruitment and tumor vascular changes are induced by low temperature, long duration (fever-like) whole body hyperthermia. J Cell Physiol.

[102] Kong G, Braun RD, Dewhirst MW (2000). Hyperthermia Enables Tumor-specific Nanoparticle Delivery: Effect of Particle Size. Cancer Res.

[103] Kirui DK, Celia C, Molinaro R (2015). Mild Hyperthermia Enhances Transport of Liposomal Gemcitabine and Improves In Vivo Therapeutic Response. Adv Healthc Mater.

[104] Li L, ten Hagen TLM, Bolkestein M (2013). Improved intratumoral nanoparticle extravasation and penetration by mild hyperthermia. J Control Release.

[105] Lammers T, Peschke P, Kühnlein R (2007). Effect of radiotherapy and hyperthermia on the tumor accumulation of HPMA copolymer-based drug delivery systems. J Control Release.

[106] Bagley AF, Scherz-Shouval R, Galie PA (2015). Endothelial Thermotolerance Impairs Nanoparticle Transport in Tumors. Cancer Res.

[107] Weinstein J, Magin R, Yatvin M, Zaharko D (1979). Liposomes and local hyperthermia: selective delivery of methotrexate to heated tumors. Science (80- ).

[108] Needham D, Anyarambhatla G, Kong G, Dewhirst MW (2000). A new temperature-sensitive liposome for use with mild hyperthermia: characterization and testing in a human tumor xenograft model. Cancer Res.

[109] Needham D, Dewhirst MW (2001). The development and testing of a new temperature-sensitive drug delivery system for the treatment of solid tumors. Adv Drug Deliv Rev.

[110] Kong G, Anyarambhatia G, Petros WP (2000). Efficacy of Liposomes and Hyperthermia in a Human Tumor Xenograft Model: Importance of Triggered Drug Release. Cancer Res.

[111] Manzoor AA, Lindner LH, Landon CD (2012). Overcoming limitations in nanoparticle drug delivery: triggered, intravascular release to improve drug penetration into tumors. Cancer Res.

[112] Yarmolenko PS, Zhao Y, Landon C (2010). Comparative effects of thermosensitive doxorubicin-containing liposomes and hyperthermia in human and murine tumours. Int J Hyperth.

[113] Hauck ML, LaRue SM, Petros WP (2006). Phase I trial of doxorubicin-containing low temperature sensitive liposomes in spontaneous canine tumors. Clin Cancer Res.

[114] Celsion A Study of ThermoDox^TM^ in Combination With Radiofrequency Ablation (RFA) in Primary and Metastatic Tumors of the Liver. https://www.clinicaltrials.gov/ct2/show/NCT00441376?term=thermodox+RFA+liver&rank=1.

[115] Celsion Phase 1/2 Study of ThermoDox With Approved Hyperthermia in Treatment of Breast Cancer Recurrence at the Chest Wall (DIGNITY). https://www.clinicaltrials.gov/ct2/show/NCT00826085?term=thermodox+rcw&rank=1.

[116] Celsion Study of ThermoDox With Standardized Radiofrequency Ablation (RFA) for Treatment of Hepatocellular Carcinoma (HCC) (OPTIMA). https://clinicaltrials.gov/ct2/show/study/NCT02112656.

[117] Master A, Livingston M, Sen Gupta A (2013). Photodynamic nanomedicine in the treatment of solid tumors: Perspectives and challenges. J Control Release.

[118] Fingar VH (1996). Vascular Effects of Photodynamic Therapy. J Clin Laser Med Surg.

[119] Fingar VH, Wieman TJ, Wiehle SA, Cerrito PB (1992). The role of microvascular damage in photodynamic therapy: the effect of treatment on vessel constriction, permeability, and leukocyte adhesion. Cancer Res.

[120] Star WM, Marijnissen HPA, Berg-Blok AE van den (1986). Destruction of Rat Mammary Tumor and Normal Tissue Microcirculation by Hematoporphyrin Derivative Photoradiation Observed in Vivo in Sandwich Observation Chambers. Cancer Res.

[121] Tate RM, Vanbenthuysen KM, Shasby DM, McMurtry IF, Repine JE (1982). Oxygen-radical-mediated permeability edema and vasoconstriction in isolated perfused rabbit lungs. Am Rev Respir Dis.

[122] Snyder JW, Greco WR, Bellnier DA, Vaughan L, Henderson BW (2003). Photodynamic therapy: a means to enhanced drug delivery to tumors. Cancer Res.

[123] Chen B, Pogue BW, Zhou X (2005). Tumor Vascular Permeabilization by Vascular-Targeting Photosensitization: Effects, Mechanism, and Therapeutic Implications. Clin Cancer Res.

[124] Cheng C, Debefve E, Haouala A (2010). Photodynamic therapy selectively enhances liposomal doxorubicin uptake in sarcoma tumors to rodent lungs. Lasers Surg Med.

[125] Mitsunaga M, Ogawa M, Kosaka N (2011). Cancer cell-selective in vivo near infrared photoimmunotherapy targeting specific membrane molecules. Nat Med.

[126] Sano K, Nakajima T, Choyke PL, Kobayashi H (2013). Markedly Enhanced Permeability and Retention Effects Induced by Photo-immunotherapy of Tumors. ACS Nano.

[127] Sano K, Nakajima T, Choyke PL, Kobayashi H (2014). The Effect of Photoimmunotherapy Followed by Liposomal Daunorubicin in a Mixed Tumor Model: A Demonstration of the Super-Enhanced Permeability and Retention Effect after Photoimmunotherapy. Mol Cancer Ther.

[128] Zhen Z, Tang W, Chuang Y-J (2014). Tumor Vasculature Targeted Photodynamic Therapy for Enhanced Delivery of Nanoparticles. ACS Nano.

[129] Wilhelm S, Tavares AJ, Dai Q (2016). Analysis of nanoparticle delivery to tumours. Nat Rev Mater.

[130] Ehling J, Theek B, Gremse F (2014). Micro-CT Imaging of Tumor Angiogenesis: Quantitative Measures Describing Micromorphology and Vascularization. Am J Pathol.

[131] Lammers T, Rizzo LY, Storm G, Kiessling F (2012). Personalized nanomedicine. Clin Cancer Res.

[132] Miller MA, Gadde S, Pfirschke C (2015). Predicting therapeutic nanomedicine efficacy using a companion magnetic resonance imaging nanoparticle. Sci Transl Med.

[133] Pérez-Medina C, Abdel-Atti D, Tang J (2016). Nanoreporter PET predicts the efficacy of anti-cancer nanotherapy. Nat Commun.

[134] Kano MR, Bae Y, Iwata C (2007). Improvement of cancer-targeting therapy, using nanocarriers for intractable solid tumors by inhibition of TGF-beta signaling. Proc Natl Acad Sci U S A.

[135] Jain RK, Stylianopoulos T (2010). Delivering nanomedicine to solid tumors. Nat Rev Clin Oncol.

[136] Miao L, Huang L (2015). Exploring the Tumor Microenvironment with Nanoparticles.

[137] Egeblad M, Nakasone ES, Werb Z (2010). Tumors as Organs: Complex Tissues that Interface with the Entire Organism. Dev Cell.

[138] Egeblad M, Rasch MG, Weaver VM (2010). Dynamic interplay between the collagen scaffold and tumor evolution. Curr Opin Cell Biol.

[139] Maeda H, Khatami M (2018). Analyses of repeated failures in cancer therapy for solid tumors: poor tumor-selective drug delivery, low therapeutic efficacy and unsustainable costs. Clin Transl Med.

[140] Stewart E, Federico SM, Chen X (2017). Orthotopic patient-derived xenografts of paediatric solid tumours. Nature.

[141] Zhou H, Qian W, Uckun FM (2015). IGF1 Receptor Targeted Theranostic Nanoparticles for Targeted and Image-Guided Therapy of Pancreatic Cancer. ACS Nano.

[142] Leong DT, Ng KW (2014). Probing the relevance of 3D cancer models in nanomedicine research. Adv Drug Deliv Rev.

[143] Cabral H, Murakami M, Hojo H (2013). Targeted therapy of spontaneous murine pancreatic tumors by polymeric micelles prolongs survival and prevents peritoneal metastasis. Proc Natl Acad Sci.

[144] Deshantri AK, Kooijmans SAA, Kuijpers SA (2016). Liposomal prednisolone inhibits tumor growth in a spontaneous mouse mammary carcinoma model. J Control Release.

[145] Zheng J, Jaffray D, Allen C (2009). Quantitative CT Imaging of the Spatial and Temporal Distribution of Liposomes in a Rabbit Tumor Model. Mol Pharm.

[146] Primeau AJ, Rendon A, Hedley D, Lilge L, Tannock IF (2005). The Distribution of the Anticancer Drug Doxorubicin in Relation to Blood Vessels in Solid Tumors. Clin Cancer Res.

[147] Ekdawi SN, Stewart JMP, Dunne M (2015). Spatial and temporal mapping of heterogeneity in liposome uptake and microvascular distribution in an orthotopic tumor xenograft model. J Control Release.

[148] De Cock I, Zagato E, Braeckmans K (2015). Ultrasound and microbubble mediated drug delivery: acoustic pressure as determinant for uptake via membrane pores or endocytosis.

[149] De Cock I, Lajoinie G, Versluis M, De Smedt SC, Lentacker I (2016). Sonoprinting and the importance of microbubble loading for the ultrasound mediated cellular delivery of nanoparticles. Biomaterials.

[150] Fan Z, Liu H, Mayer M, Deng CX (2012). Spatiotemporally controlled single cell sonoporation. Proc Natl Acad Sci.

[151] Roovers S, Segers T, Lajoinie G (2019). The Role of Ultrasound-Driven Microbubble Dynamics in Drug Delivery: From Microbubble Fundamentals to Clinical Translation.

[152] Norum O-J, Selbo PK, Weyergang A, Giercksky K-E, Berg K (2009). Photochemical internalization (PCI) in cancer therapy: From bench towards bedside medicine. J Photochem Photobiol B Biol.

[153] Alizadeh D, Zhang L, Hwang J-, Schluep T, Badie B (2010). Tumor-associated macrophages are predominant carriers of cyclodextrin-based nanoparticles into gliomas. Nanomedicine Nanotechnology, Biol Med.

[154] Maeda H, Wu J, Sawa T, Matsumura Y, Hori K (2000). Tumor vascular permeability and the EPR effect in macromolecular therapeutics: a review. J Control Release.

[155] Sykes EA, Chen J, Zheng G, Chan WCW (2014). Investigating the Impact of Nanoparticle Size on Active and Passive Tumor Targeting Efficiency. ACS Nano.

[156] Huynh E, Leung BYC, Helfield BL (2015). In situ conversion of porphyrin microbubbles to nanoparticles for multimodality imaging. Nat Nanotechnol.

[157] Pellow C, Goertz DE, Zheng G (2017). Breaking free from vascular confinement: status and prospects for submicron ultrasound contrast agents.

